# Algal Autophagy Is Necessary for the Regulation of Carbon Metabolism Under Nutrient Deficiency

**DOI:** 10.3389/fpls.2020.00036

**Published:** 2020-02-05

**Authors:** Masataka Kajikawa, Hideya Fukuzawa

**Affiliations:** Graduate School of Biostudies, Kyoto University, Kyoto, Japan

**Keywords:** autophagy, *Chlamydomonas*, lipid, nutrient deficiency, starch

## Abstract

Autophagy is a mechanism to recycle intracellular constituents such as amino acids and other carbon- and nitrogen (N)-containing compounds. Although autophagy-related (ATG) genes required for autophagy are encoded by many algal genomes, their functional importance in microalgae in nutrient-deficiency has not been appraised using *ATG*-defective mutants. Recently, by characterization of an insertional mutant of the *ATG8* encoding a ubiquitin-like protein indispensable for autophagosome formation in a green alga *Chlamydomonas reinhardtii,* we have provided evidence that supports the following notions. ATG8 protein is required for the degradation of lipid droplets and triacylglycerol (TAG) triggered by resupply of N to cell culture in N-deficient conditions. ATG8 protein is also necessary for starch accumulation under phosphorus-deficient conditions. Algal autophagy is not necessary for inheritance of chloroplast and mitochondrial genomes. In this review, we discuss the physiological roles of algal autophagy associated with nutrient deficiency revealed by the genetic and biochemical analyses using disruption mutants and reagents that inhibit the fatty acid biosynthesis and vacuolar H^+^-ATPase.

## Introduction

Macroautophagy (hereafter autophagy) is a recycling system for degradation of cytoplasmic constituents in the vacuole or lysosomes (Mizushima et al., 2011; Liu and Bassham, 2012; Yang and Bassham, 2015). This pathway is conserved among eukaryotes (Liu and Bassham, 2012; Yang and Bassham, 2015). In order to understand the physiological functions of plant autophagy, the following strategies has been conducted, because the processes of autophagy, from autophagosome formation to degradation of target cellular components in the vacuole are very rapid (Yoshimoto, 2012): 1) analyzing the phenotype of autophagy-defective core *ATG* gene mutants (Doelling et al., 2002; Hanaoka et al., 2002; Yoshimoto et al., 2004; Thompson et al., 2005); 2) visualizing subcellular localization of ATG8 protein with a fluorescent protein (Yoshimoto et al., 2004; Contento et al., 2005; Merkulova et al., 2014); and 3) artificially halting the autophagic flux in the vacuole by treatment with concanamycin A, which inhibits H^+^-ATPase and thus inactivating the vacuolar acid hydrolases, leading to the autophagic body accumulating inside the vacuole (Yoshimoto et al., 2004).

The following findings have been reported regarding the regulation of the accumulation of photosynthetic assimilation products, such as lipids and starch, by autophagy. Firstly, starch synthesized in leaves by photosynthesis during the day is degraded at night, and phenotypic analysis of *atg* mutants in *Arabidopsis thaliana* suggested that autophagy facilitates this process (Wang et al., 2013). In addition, autophagy was shown to promote triacylglycerol (TAG) degradation under C-deficient conditions using the seedlings of *A. thaliana atg* mutants (Avin-Wittenberg et al., 2015). Recently, Fan et al. (2019) reported that basal autophagy is required for TAG biosynthesis by lipid turnover providing fatty acids from organellar membrane lipids in *A. thaliana*. Similarly, studies on *atg* mutants in rice which showed male sterility concluded that autophagy is necessary for TAG and starch accumulation and lipid droplet formation as well as normal reproductive post-meiotic anther development during pollen maturation (Kurusu et al., 2014). However, studies of plant autophagy have been limited to a few model species for which *atg* mutants are available, and the role of autophagy in the metabolism of carbon (C)-assimilation products throughout the plant kingdom needs to be further investigated. Because in algae the role of autophagy in the accumulation and metabolism of photosynthetic assimilation products remained unclear, studies using autophagy-deficient mutant strains have been considered necessary in addition to those using wild type cells treated with autophagy-inhibiting chemicals. In this review, the physiological functions of algal autophagy in response to nutrient deficiency will be discussed, based on recent reports of autophagy-defective mutants in *Chlamydomonas reinhardtii* (hereafter *Chlamydomonas*) and the effects of treatment of the wild-type *Chlamydomonas* cells with cerulenin and concanamycin A for inhibition of fatty acid synthesis and vacuolar lysosomal function, respectively.

### Algal Autophagy

Unicellular algae known as microalgae are photosynthetic eukaryotes, classified as one of protists. For the purpose of biofuel production, many microalgae have been nominated by screening, which accumulate high levels of C-storage compounds such as lipid and starch. Especially understanding the physiological culture conditions and the molecular mechanisms for accumulation of their lipid and starch is necessary to achieve realistic biofuel production. So far, it is reported that many algae accumulate these C-storage compounds in cells when exposed to nutrient-deficient stress conditions such as nitrogen (N)-deficiency after stopping their growth in these stress conditions but they do not die immediately and that instead, they maintain cell viability for a period of time. This cell survival under nutrient-deplete conditions suggests that autophagy is involved in the system for keeping the cell viability. Although autophagy is involved in the regulation of C metabolism in yeast, animals, and terrestrial plants, contribution of autophagy to lipid and starch metabolism in algae has not been fully understood. In several species of algae from Chlorophyta, Rhodophyta, and Chromalveolata, orthologs for known *ATG* genes have been found in genome databases, except for red algae whose genomes lack the *ATG* gene (Díaz-Troya et al., 2008a; Avin-Wittenberg et al., 2012; Jiang et al., 2012; Shemi et al., 2015). However, no autophagy-defective mutant has been reported in any species of algae so far.

In a model photosynthetic single-cell eukaryote, *Chlamydomonas*, a series of studies have been reported to provide molecular evidence of autophagy and autophagic activities responding to rapamycin, an inhibitor of the target of rapamycin (TOR) protein kinase (Pérez-Pérez et al., 2010), abiotic stresses such as endoplasmic reticulum (ER) stress, oxidative stress (Pérez-Pérez et al., 2010; Pérez-Martín et al., 2014), and metal toxicity (Pérez-Martín et al., 2015), and the following nutrient deficiency; N-deficiency (Pérez-Pérez et al., 2010; Goodenough et al., 2014), C-deficiency (Pérez-Pérez et al., 2010), and carotenoid deficiency (Pérez-Pérez et al., 2012). In the all mentioned stress conditions, the abundance of ATG8 protein and its PE-conjugated/lipidated form increases. Accumulation of ATG8 and ATG3 proteins increased in a conditional repression line of a chloroplast protease ClpP1, implying that chloroplast proteolysis systems and autophagy in *Chlamydomonas* partially complement each other (Ramundo et al., 2014). Furthermore, *in vitro* assays using recombinant ATG8 and ATG4 proteins and complementation tests with the corresponding yeast mutants revealed that correspond to the orthologs for *ATG8* and *ATG4* genes in *Chlamydomonas*, respectively, encode proteins with functions similar to those in other organisms (Pérez-Pérez et al., 2010; Pérez-Pérez et al., 2016).

Recently, we reported the functional importance of ATG8 and ATG3 proteins by using insertion mutants defect in corresponding genes in *Chlamydomonas* (Kajikawa et al., 2019). Using these autophagy-defective strains, contribution of autophagy on cell processes including photosynthesis, metabolism, and reproduction under various nutrient-deficient conditions was clarified.

### Algal Autophagy in TOR Signaling

The TOR kinase complex 1 (TORC1) is reported to be an important regulatory factor making balance between growth and autophagy in the eukaryotes (Noda and Ohsumi, 1998; Pattingre et al., 2008; Liu and Bassham, 2010). When nutrients are abundantly supplied to the cell, TOR is activated and promotes protein synthesis and cell growth, while negatively regulating autophagy by inhibiting the formation of the ATG1 complex (Mizushima et al., 2011). Land plants have TOR-dependent and TOR-independent pathways for regulation of autophagy (Pu et al., 2017). Activation of autophagy in nutrient deficiency, salt, and osmotic stress requires suppression of TOR. On the other hand, induction of autophagy under oxidative stress and ER stress conditions is suggested to be controlled independently by TOR (Pu et al., 2017; Soto-Burgos et al., 2018). Although there are still many unknown regarding algal TOR systems, it is reported that TOR, LST8 and Raptor genes, which are components of TORC1 protein complex, are widely conserved in algal genomes (Shemi et al., 2015).

**Figure 1:**
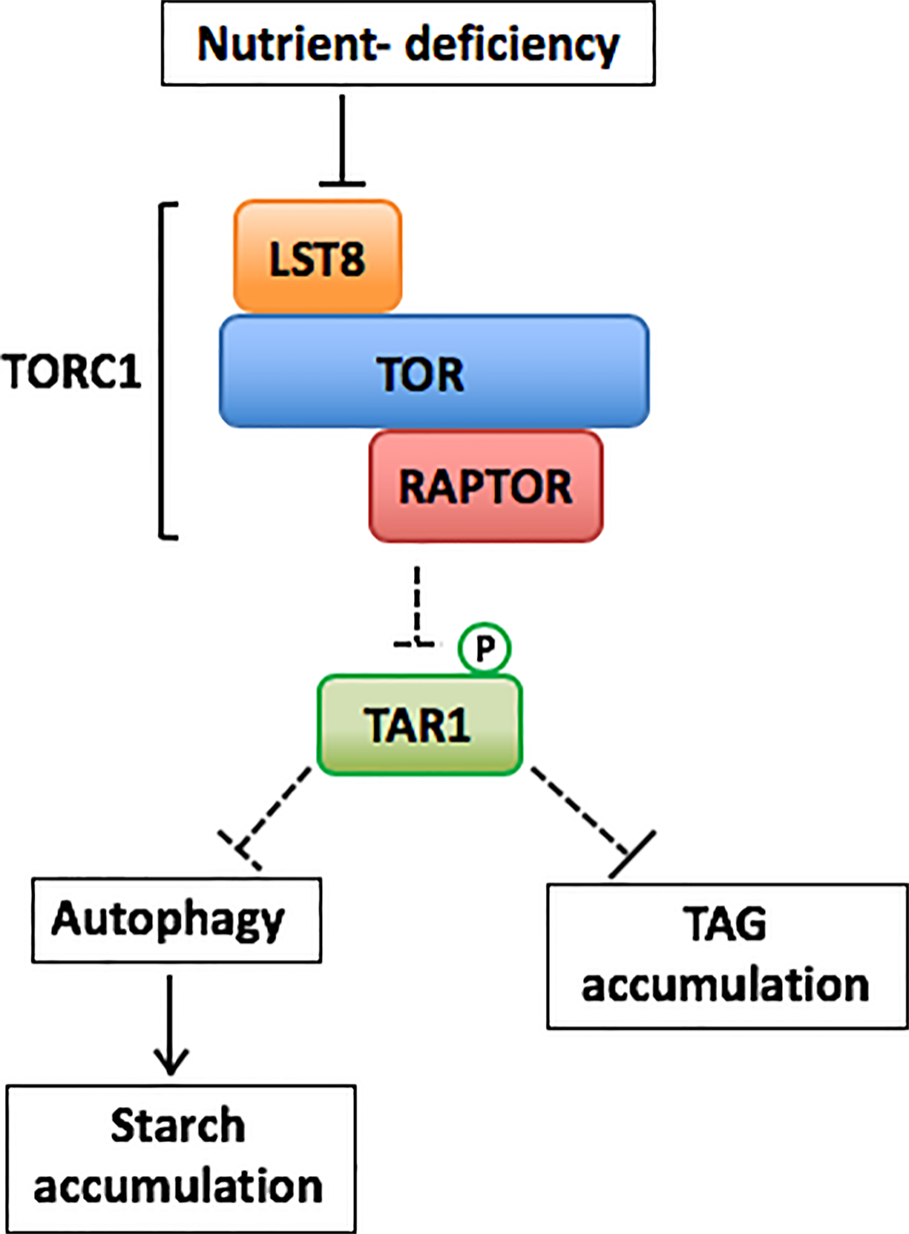
One of the possible models for TORC1-mediated regulation of autophagy and carbon metabolism in *Chlamydomonas* in response to nutrient deficiency based on models by Forzani et al. (2019); Couso et al. (2020) and Shinkawa et al. (2019). Unidentified but possible regulatory steps are shown by dashed arrows.

*Chlamydomonas* has all the components of the TORCl complex (Crespo et al., 2005; Díaz-Troya et al., 2008b). The arrest of cell cycle, bleaching, and vacuolization have been shown to be proceeded by the addition of rapamycin to the *Chlamydomonas* cells, indicating that *Chlamydomonas* has a rapamycin-sensitive TOR signaling network (Crespo et al., 2005). Addition of rapamycin also increases the expression of ATG8, led to detection of lipidated ATG8, and altered the subcellular localization of ATG8, suggesting that the rapamycin-sensitive TOR signaling network regulates autophagy in *Chlamydomonas* (Pérez-Pérez et al., 2010; Pérez-Pérez et al., 2017). Recently, Mubeen et al. (2018) demonstrated that rapamycin-induced TOR inhibition increases *de novo* amino acid synthesis due to upregulated ammonium assimilation in *Chlamydomonas*. It has been also reported that TOR inhibition by addition of TOR inhibitors, such as rapamycin, AZD8055, or Torin1, activates expression of genes encoding TAG biosynthesis-related enzymes and increased TAG accumulation in *Chlamydomonas* and a red alga *Cyanidioschyzon merolae* (Imamura et al., 2015; Imamura et al., 2016). Furthermore, it has been suggested that TOR in *Chlamydomonas* regulates the biosynthesis of the signaling molecules, inositol polyphosphates (InsPs) (Couso et al., 2016). A mutant, *vip1-1*, of the inositol polyphosphate kinase (*VIP1*) gene involved in the biosynthesis of the InsPs, showed hypersensitivity to rapamycin and increased TAG accumulation even under nutrient replete conditions (Couso et al., 2016). In addition, these reports indicate an interaction between TOR signaling network and lipid metabolism in algae (Pérez-Pérez et al., 2017). Most recently, through the characterization of a hypomorphic mutant of the *LST8* gene, *lst8-1* under P deficiency, Couso et al. (2020) demonstrated that P deficiency reduces the abundance of LST8 proteins and downregulates activity of TORC1 to activate autophagy and TAG synthesis in *Chlamydomonas* (**Figure 1**). In the N-deficient conditions, any significant phenotype was not observed in the *lst8-1* mutant.

Despite these energetic studies in this field, any direct molecular evidence that algal autophagy is involved in interaction between TOR signaling network and lipid metabolism has not been provided and needs to be examined experimentally in future. By quantitative phosphoproteomic approach, total 258 of phosphosites from 219 unique phosphopeptides were detected in *Chlamydomonas* wild-type cells as significantly modulated by inhibition of TOR (Werth et al., 2019). The key target factors downstream of TOR that are involved in autophagy induction and regulation of lipid turnover and link each other might be discovered from proteins composed of such phosphopeptides.

### Role of Algal Autophagy in Carbon Metabolism

*Chlamydomonas* accumulates high levels of neutral lipids and starch in the absence of nutrients such as N, P, and sulfur (S). In addition, the *Chlamydomonas* genome contains *ATG* genes related to autophagy. Therefore, we attempted to isolate mutants that lack the *ATG8* and *ATG3* genes, which are required for the formation of autophagosomal membranes—an early process of autophagy (Kajikawa et al., 2019). For both genes, only one copy was present in the *Chlamydomonas* genome, and we predicted that their deletion would lead to an autophagy defect. DNA tag-insertion mutants of each gene were selected, one by one, from approximately 4,600 mutant strain libraries, and antibodies were used to show that these were functionally deficient mutants. In the *atg8* and *atg3* mutants, the cell viability under N-deficient conditions was significantly reduced. After two weeks, the survival rate of the wild-type cells was 80%, whereas the survival rate in both mutant strains decreased to less than 5%, indicating that autophagy is necessary for algal cell survival.

The role of autophagy in the control of C metabolic processes was determined by examining the accumulation of neutral lipids (TAG) and starch under nutrient-deficient conditions, using *atg8* mutants as a representative strain. Under N- and S-deficient conditions, the *atg8* mutant accumulated starch in the same way as did the wild-type strain during the early stress phase. However, since the accumulated starch in the *atg8* mutant was broken down after the second day of stress, this suggests that autophagy was required to maintain the accumulated starch content. In addition, when the medium was replaced with N-deficient conditions before being returned to the N-replete condition, TAG and starch degraded rapidly in the wild-type strain, while degradation of TAG and oil droplets in the *atg8* cells occurred approximately 6 h later than in the wild strain. However, the rate of starch degradation in the *atg8* mutant was unaffected, suggesting that autophagy is required for rapid degradation of TAG.

The green algae accumulated starch and TAG under P-deficient conditions, as well as under N- and S-deficient conditions. However, although the *atg8* mutants accumulated TAG at rates similar to that of the wild-type strain, starch accumulation did not occur from the beginning of the deficiency period, suggesting that autophagy was required for starch accumulation in response to P deficiency (**Figure 1**).

Cellular organelles, such as chloroplasts and mitochondria, have their own genomes, and only the genomes from one parent are inherited during mating—a process known as maternal inheritance. The *Chlamydomonas* cell undergoes a shift from vegetative growth to reproductive growth, and gametogenesis occurs meiotically under N-deficient conditions. Because autophagy is involved in the maternal inheritance of mitochondria in mice (Al Rawi et al., 2011) and nematode (Sato and Sato, 2011), experiments were conducted using the *atg8* mutant to determine whether autophagy was involved in the maternal inheritance of algae. The results showed that maternal inheritance continued as normal during mating of both mitochondrial genomes, and that algal autophagy is not involved in maternal inheritance.

### Revealing the Relationship Between Algal Autophagy and Lipid Metabolism by Adding Inhibitors

Heredia-Martínez et al. (2018) reported that chloroplast damage in *Chlamydomonas* cells induced by the addition of the fatty acid biosynthesis inhibitor, cerulenin, triggers autophagy. In addition, Couso et al. (2018) reported that changes in cells were suppressed when the protein degradation system was suppressed by treating the cells with concanamycin A to stop vacuole flux. The advantage of using such inhibitors is to be able to avoid downstream effects and to examine the effects on cells when a particular *in vivo* pathway is inhibited by time-limited conditions. In both cases, the addition of the inhibitor increased the expression of the ATG8 protein, which suggested autophagy was activated. When cerulenin was added to the cells, chloroplast shrinkage and thylakoid membrane aggregation was observed along with a decrease in photosynthetic proteins and ribosomal proteins, and a decrease in maximum quantum yield. Furthermore, the amount of ROS increased, while the thylakoid membrane lipid, MGDG, decreased. Both qRT-PCR and RNAseq analyses showed that the expression of genes involved in protein degradation, stress response, and signal transduction were altered.

Heredia-Martínez et al. (2018) have proposed a model demonstrating that when fatty acid synthesis is inhibited by cerulenin, the amount of MGDG is reduced, thereby impairing chloroplast function and causing thylakoid membrane aggregation and generation of ROS. This chloroplast damage propagates up to the nucleus as a retrograde signal and causes activation of autophagy with changes in gene expression, as well as the activation of proteolytic systems. Future research will be necessary to further verify the time-based vertical relationship and causality of these multiple-response reactions. In addition, by comparing the effect of treatment with cerulenin between the wild-type strain and the autophagy-deficient strain, it is possible to verify to what extent autophagy affects these chloroplast injuries.

Couso et al. (2018) reported that development of lipid droplets and TAG accumulation under N- or P-deficient conditions were repressed when the lytic digestion in the vacuolar or lysosomal lumen was inhibited in *Chlamydomonas* WT cells after treatment with concanamycin A, an inhibitor of vacuolar H^+^-ATPase activity. Moreover, treatment with concanamycin A during these nutrient-deficient periods suppressed the degradation of ribosomal proteins such as RPS6 and RPL37. This suggested that concanamycin A inhibits autophagic flux (Couso et al., 2018). By the live-cell imaging using the *Chlamydomonas* transgenic line expressing red fluorescent protein (mCherry)-tagged ATG8, the interactions and fusion between mCherry-labeled structures and lipid droplets were observed in the N deficiency (Tran et al., 2019). Based on this observation, Tran et al. (2019) suggests that autophagy-related pathway might be involved in lipid droplet turnover in the alga. On the other hand, genetic inhibition of autophagy by knockout of the core *ATG* genes compromises mobilization of various cytoplasmic components into the vacuolar or lysosomal lumen (Kajikawa et al., 2019). The *atg* mutants formed lipid droplets and accumulated TAG under both N- and P-deficient conditions as the WT cells did. In the red alga *C. merolae* which do not have any core *ATG* gene, mRNA abundance of genes encoding glycerol-3-phosphate acyltransferase (GPAT) and acyl-CoA:diacylglycerol acyltransferase (DGAT)in TAG biosynthesis and the level of TAG content increase under TOR-inhibition condition (Imamura et al., 2015). These reports suggest that TOR signaling regulates development of lipid droplets and TAG accumulation without though activation of autophagic pathway in the algae (**Figure 1**).

Interestingly, a dual-specificity tyrosine phosphorylation-regulated kinase, TAG accumulation regulator 1 (TAR1) has been reported to be necessary for the degradation of chlorophyll and photosynthesis-related proteins and acetate-dependent TAG accumulation in photomixotrophic N-deficient conditions (Kajikawa et al., 2015). In addition, in photoautotrophic N-deficient conditions, the *tar1*-defective mutant maintained higher levels of cell viability, TAG, and starch with lower accumulation of hydrogen peroxide compared with those of wild type (WT) cells with bubbling of air containing 5% carbon dioxide (Shinkawa et al., 2019). Recently, Forzani et al. (2019) and Barrada et al. (2019) reported that TOR represses an *A. thaliana* TAR1 ortholog, YAK1 to promote meristem activity and plant growth. YAK1 interacts with RAPTOR and is directly phosphorylated by TOR complex in *A. thaliana* (Forzani et al., 2019). These findings open a possibility that the *Chlamydomonas* TAR1 is also directly regulated by TOR-mediated phosphorylation (**Figure 1**). Relationship of the *Chlamydomonas* TAR1 kinase and regulation of algal autophagy and TOR signaling network could further deepen the understanding of the regulatory mechanism of cellular responses to nutrient-deplete stress conditions. Especially analyses of protein-protein interactions among regulatory kinases and autophagy components could contribute to better understanding of autophagy-related processes in photosynthetic organisms.

## Concluding Remarks and Future Perspectives

The research into autophagy-defective mutants and the effects of inhibitor addition have clarified the role of algal autophagy in the accumulation and degradation of neutral lipids and starch under various nutritional conditions. In the future, by combining these approaches—i.e. by adding rapamycin, cerulenin, concanamycin A, and other inhibitors of a specific pathway to the autophagy-defective mutants, and examining the resultant time-dependent phenotypic changes—we can verify the mechanism that works in parallel with autophagy, and which plays a role in complementing autophagy in algae.

## Author Contributions

MK and HF wrote this manuscript.

## Funding

This work was supported by JSPS KAKENHI grant number 16H04805 (to HF), 17K07753 (to MK), JST ALCA program JPMJAL1105 (to HF), and NEDO project number P10010 (to HF).

## Conflict of Interest

The authors declare that the research was conducted in the absence of any commercial or financial relationships that could be construed as a potential conflict of interest.
